# Inhibition of interaction between *Staphylococcus aureus* α-hemolysin and erythrocytes membrane by hydrolysable tannins: structure-related activity study

**DOI:** 10.1038/s41598-020-68030-1

**Published:** 2020-07-07

**Authors:** Ewa Olchowik-Grabarek, Szymon Sekowski, Maciej Bitiucki, Izabela Dobrzynska, Vadim Shlyonsky, Maksim Ionov, Paweł Burzynski, Anna Roszkowska, Izabela Swiecicka, Nodira Abdulladjanova, Maria Zamaraeva

**Affiliations:** 1 Department of Microbiology and Biotechnology, Laboratory of Molecular Biophysics, Faculty of Biology, University of Bialystok, Konstanty Ciolkowski Street 1J, 15-245 Białystok, Poland; 2 Laboratory of Bioanalysis, Faculty of Chemistry, University of Bialystok, Konstanty Ciolkowski Street 1K, 15-245 Białystok, Poland; 3 Department of Physiology and Pharmacology, Université libre de Bruxelles, Route de Lennik 808, CP604, 1070 Brussels, Belgium; 4 Department of General Biophysics, Faculty of Biology and Environmental Protection, University of Lodz, Pomorska Street 141/143, 90-236 Lodz, Poland; 5 Department of Microbiology and Biotechnology, Laboratory of Applied Microbiology, Faculty of Biology, University of Bialystok, Konstanty Ciolkowski Street 1J, 15-245 Białystok, Poland; 6 Institute of Bioorganic Chemistry, Academy of Sciences of the Republic of Uzbekistan, Abdullaev Street 83, 100143 Tashkent, Uzbekistan

**Keywords:** Biophysics, Molecular biology

## Abstract

The objective of the study was a comparative analysis of the antihemolytic activity against two *Staphylococcus aureus* strains (8325-4 and NCTC 5655) as well as α-hemolysin and of the membrane modifying action of four hydrolysable tannins with different molecular mass and flexibility: 3,6-bis-*O*-di-*O*-galloyl-1,2,4-tri-*O*-galloyl-*β*-d-glucose (T1), 1,2,3,4,5-penta-*O*-galloyl-*β*-d-glucose (T2), 3-*O*-galloyl-1,2-valoneoyl-*β*-d-glucose (T3) and 1,2-di-*O*-galloyl-4,6-valoneoyl-*β*-d-glucose (T4). We showed that all the compounds studied manifested antihemolytic effects in the range of 5–50 µM concentrations. However, the degree of the reduction of hemolysis by the investigated tannins was not uniform. A valoneoyl group—containing compounds (T3 and T4) were less active. Inhibition of the hemolysis induced by α-hemolysin was also noticed on preincubated with the tannins and subsequently washed erythrocytes. In this case the efficiency again depended on the tannin structure and could be represented by the following order: T1 > T2 > T4 > T3. We also found a relationship between the degree of antihemolytic activity of the tannins studied and their capacity to increase the ordering parameter of the erythrocyte membrane outer layer and to change zeta potential. Overall, our study showed a potential of the T1 and T2 tannins as anti-virulence agents. The results of this study using tannins with different combinations of molecular mass and flexibility shed additional light on the role of tannin structure in activity manifestation.

## Introduction

Over the past few years, a significant increase in bacterial resistance to antibiotics and transference of resistance genes from animal to human strains has become a global medical problem. Therefore, constant search for new antimicrobial agents among them being compounds of plant origin, including polyphenols, is ongoing^1,2^.

In addition to the antibiotic approaches of combating bacteria, anti-virulence strategies have also been considered recently. An anti-virulent strategy assumes a direct effect of compounds on virulent factors by reducing their production or neutralizing their activity without affecting bacterial growth. It is assumed that this approach allows to reduce or avoid drug-resistance development^3,4^.

**Figure 1:**
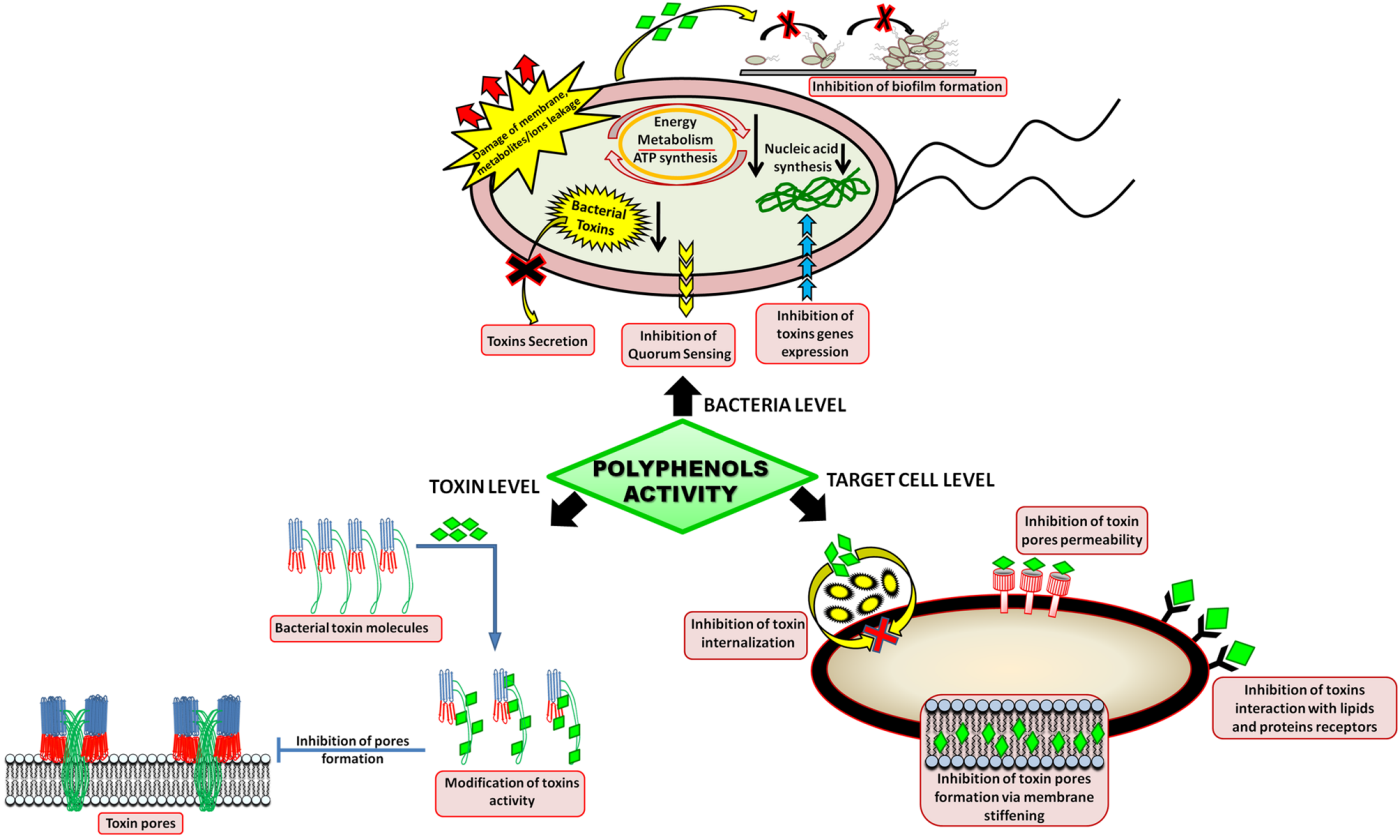
Mechanisms of polyphenols protective action against bacterial activity on three different levels: (1) direct affecting bacterial cell biology (bacteria level), (2) modifying properties of secreted toxins (toxin level), (3) increasing target cells resistance (target cell level).

The antibacterial activity of polyphenols is realized at the level of whole bacteria through the damage (modification) of the membrane structure^5–7^ and via inhibition of energy metabolism^8^, production or secretion of toxins^9–12^, as well as by prevention of biofilm formation^13,14^ (Fig. 1).

Polyphenols can also directly interact with the released toxins, thus modifying their structure and activity^15–21^. Moreover, polyphenols can act at the level of target cells, increasing their resistance to toxins (Fig. 1). The studies of Olchowik-Grabarek et al.^22^ showed that the antihemolytic activity of Sumac tannins against *Bacillus cereus* cytolysins was associated with stiffening of the erythrocyte membrane. Another example is the study of the Morinaga’s group^23^, which found that resveratrol inhibited the internalization of cholera toxin by stiffening the membranes of Vero kidney cells. It was shown that polyphenols of *Berberis aristata* and *Camellia sinensis* inhibited the hemagglutination activity of carbapenem-resistant *Escherichia coli* via interaction with erythrocytes receptors^24^. Reducing the ability of the listeriolysin O (*Listeria monocytogenes*) to bind to phagosome cholesterol has been shown for galloylated catechins^25^.

Furthermore, direct blocking the channels formed by pore-forming toxins in the host cells membranes was shown in the presence of polyphenol compounds. For example, polyphenols were demonstrated to inhibit the anion-selective, urea permeable channel formed by VacA, a vacuolating cytotoxin produced by *Helicobacter pylori*^26^ and a pore formed by *Staphylococcus aureus* α-hemolysin (αHL)^27^.

*S. aureus* is one of the most widespread microorganisms in nature and human-associated environments^28,29^, and also the most dangerous representative of the genus *Staphylococcus.* This bacterium causes a broad spectrum of diseases both in humans and animals, e.g. skin diseases (folliculitis, furunculosis, dermatitis) and invasive diseases such as pneumonia, osteomyelitis and sepsis as well as many others, including food poisoning^30^. *S. aureus* is Gram-positive bacterium whose characteristic feature is the presence of staphyloxanthin, a carotenoid pigment that gives these bacteria a golden colour of their colonies and exhibits antioxidant properties, protecting them from reactive oxygen species (ROS) produced by neutrophils^31^.

The toxicity of *S. aureus* strains originates from multiple virulence factors, present on the surface of the bacterial cell as well as secreted outside, which are associated with specific diseases^30^. The virulence factors include those responsible for adhesion, invasion and colonization, factors that inhibit the host’s immune response and toxins, such as: receptor—mediated toxins (the toxic shock syndrome toxin—TSST-1, enterotoxins), exfoliative toxins (serine protease) and cytolysins. *S. aureus* produces cytolysins (leukocidins and hemolysins) that are bi-component toxins like gamma toxin (HlgAB, HlgCB), LUK (LukED, LukAB), Panton-Valentine Leukocidin (PVL), and a monocomponent toxin like αHL. All the above cytolysins are called pore—forming toxins (PFTs) because they form pores of different sizes in the host cell membrane. PFTs are divided into two families according to the secondary structure of the transmembrane region in the pore structure, α-helical PFTs (α-PFTs) and β-barrel PFTs (β-PFTs). The above-mentioned pore-forming toxins belong to β-PFTs^32^. Cytolysins also include β-hemolysin (sphingomyelinase) and δ-toxin (PSM-phenol-soluble modulins, types α and β)^33^, which can act synergistically, causing cell lysis^34^.

Among the polyphenols, tannins are of great interest as antibacterial compounds due to their ability to interact strongly with proteins, lipids, and polysaccharides^3,35,36^.

Based on the structure, this class of compounds is divided into three groups: (1) hydrolysable tannins that are esters of sugar and phenol carbonic acids, (2) condensed tannins that are polymers of flavan-3-ol, and (3) complex tannins*.* Hydrolysable tannins are general derivatives of gallic acid (gallotannins) or hexahydroxydiphenilic acid (ellagitannins)^37,38^. Ellagitannins can undergo oxidative coupling reactions, leading to the formation of tannins with valoneoyl groups in their structure^39,40^. Some authors attribute gallocatechins and their gallates to the tannin group since they are ester catechins with gallic acid, a monomer of hydrolysable tannins^41^.

The activity of tannins depends on their structure and is determined not only by molecular mass (MM) and the number of hydroxyl and aromatic rings but also on their location in the molecule, which affects their molecular bulk, flexibility and hydrophobicity, and ultimately their biological effect^42–46^.

However, it should be noted that presently no strict correlation has been established between all these parameters. And there is a lot of contradicting data. For example, it was shown that gallotannins having a larger MM inhibit protein kinase C weaker than ellagitannins, indicating the important role of a hexahydroxydiphenoyl group in the manifestation of this activity^47^. On the other hand, it was shown that the presence of free galloyl groups in the molecule of ellagitannins increases their ability to bind to albumin^45^. In addition, the structure of proteins also plays an important role in the interaction of tannin with proteins^42,48^. Not all tannins show a positive correlation between MM and their biological effect. For instance, it was shown that tannins with a larger MM interact weaker with staphylococcal enterotoxin^49^.

Therefore in the present work, a comparative study of the antihemolytic activity against two *S. aureus* strains as well as αHL and the erythrocyte membrane modifying action of four hydrolysable tannins was conducted. These tannins have different paired combinations of features (MM and flexibility due to the presence of valoneoyl groups), thus, the results of this study shed additional light on the role of tannin structure in activities manifestation.

## Results

### Antihemolytic activity and antimicrobial effect of tannins

**Figure 2:**
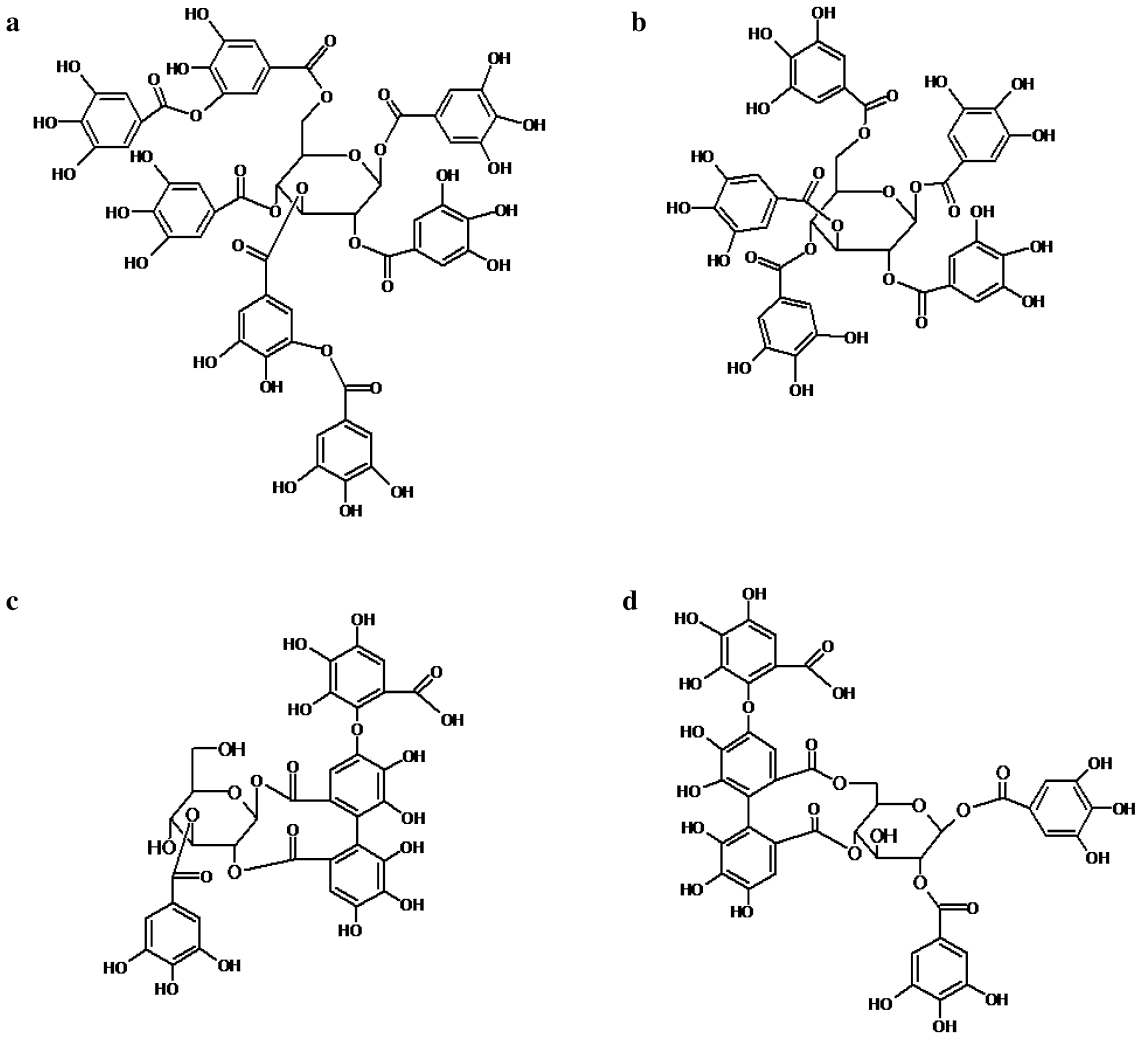
Chemical structure of 3,6-bis-*O*-di-*O*-galloyl-1,2,4-tri-*O*-galloyl-β-d-glucose (T1) (**a**), 1,2,3,4,5-penta-*O*-galloyl-β-d-glucose (PGG) (T2) (**b**), 3-*O*-galloyl-1,2-valoneoyl-β-d-glucose (T3) (**c**), and 1,2-di-*O*-galloyl-4,6-valoneoyl-β-d-glucose (T4) (**d**).

In this work we studied the protective effects of four hydrolysable tannins (3,6-bis-*O*-di-*O*-galloyl-1,2,4-tri-*O*-galloyl-*β*-d-glucose (T1) and 1,2,3,4,5-penta-*O*-galloyl-*β*-d-glucose (PGG) (T2), as well as 3-*O*-galloyl-1,2-valoneoyl-*β*-d-glucose (T3) and 1,2-di-*O*-galloyl-4,6-valoneoyl-*β*-d-glucose (T4) (Fig. 2) containing a valoneoyl group at C1 and C2 positions (T3) and at C4 and C6 ones (T4) on hemolysis caused by secreted toxins of two *S. aureus* strains*,* 8325-4 and NCTC 5655, and by pure αHL. The *S. aureus* strains used in the study differ in production of cytolysins. While 8325-4 produces α-, β-, δ- and γ-hemolysin^50^, NCTC 5655 secretes only αHL^51^. The hemolytic activity of the *S. aureus* strains was evaluated on sheep erythrocytes which show high sensitivity to αHL^52^.

**Figure 3:**
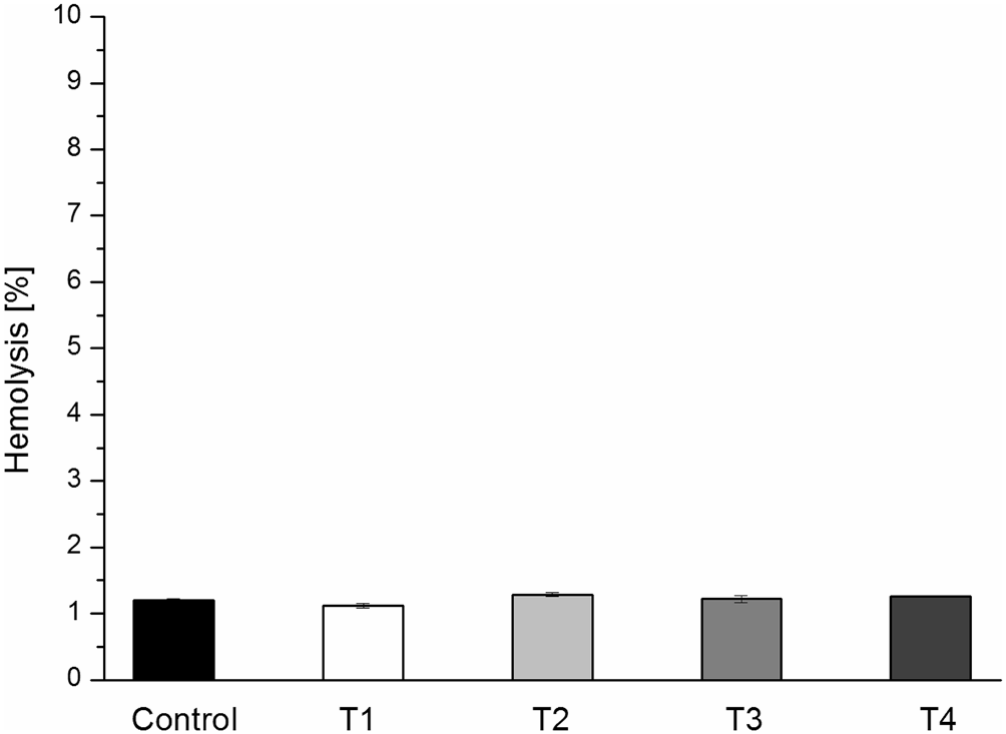
Percentage of hemolysis of sheep erythrocytes in presence studied tannins at the concentration of 50 μM (T1, T2, T3 and T4).

At first we examined if studied tannins induce hemolysis of sheep erythrocytes in the concentration range 1–50 µM. The obtained results showed that even at the highest concentration (50 µM) percentage of hemolysis was at a level as control and amounted to a little more than 1% (Fig. 3). These studied indicated that tested tannins do not induce hemolysis.

**Figure 4:**
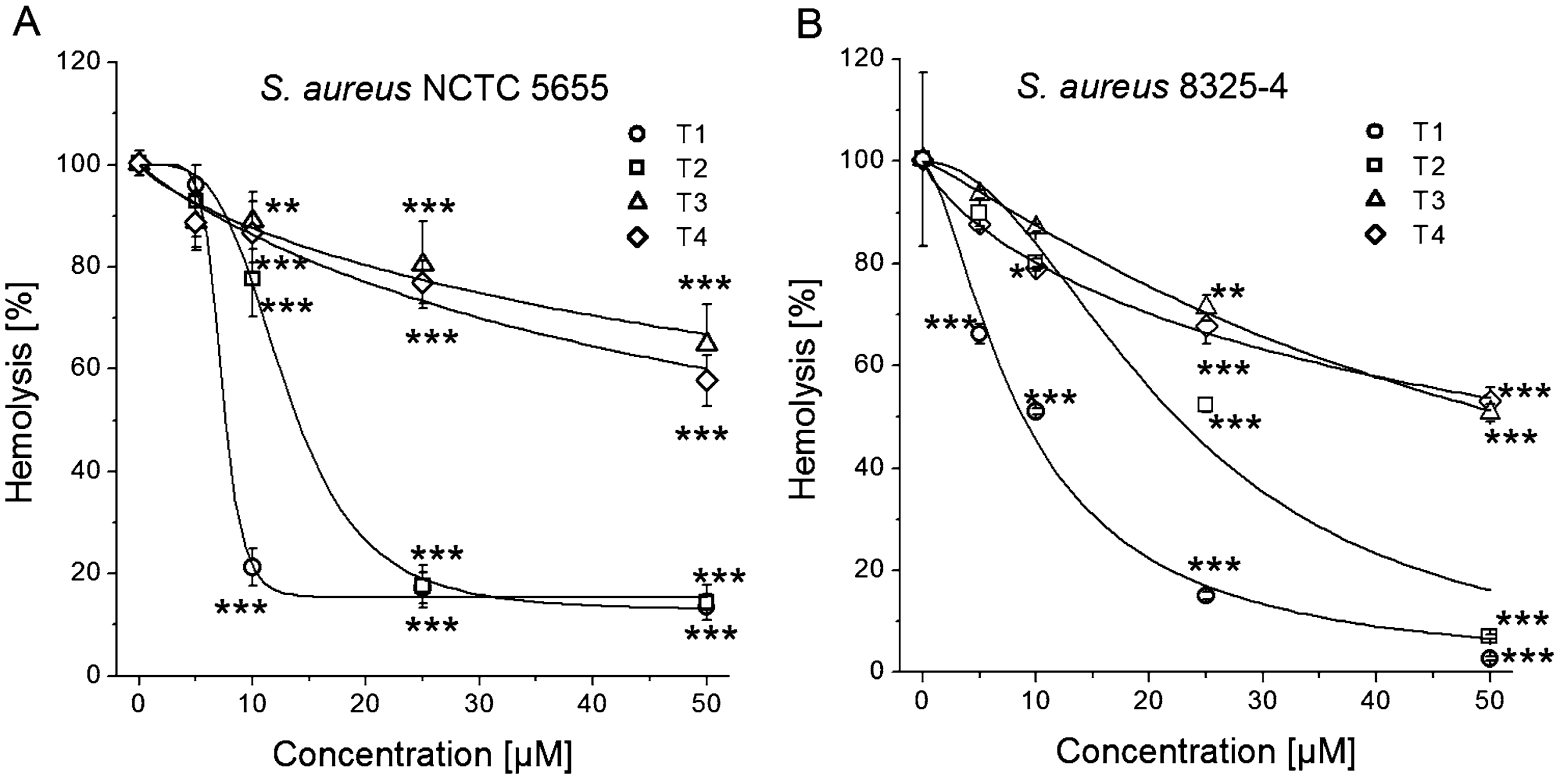
Protective effect of tannins (T1, T2, T3, and T4) against hemolysis induced by incubation of sheep erythrocytes with *S. aureus* NCTC 5655 (**A**) and *S. aureus* 8325-4 (**B**). The data presented are the means ± SD, n = 6. Lines represent best fit of logistic equation to the data. The effects of the tannins were statistically significant according to one-way ANOVA test, * < 0.05, **p < 0.01, ***p < 0.001.

The next we studied the protective effects of tested tannins on hemolysis induced by *S. aureus* strains. The exposure of erythrocytes to NCTC 5655 cells resulted in 61.16 ± 1.47% hemolysis, while in the presence of the 8325-4 cells erythrocytes underwent 71.88 ± 10.35% hemolysis. These numbers were taken as 100% for each series of the experiments. Figure 4 shows that tannins protect erythrocytes from hemolysis in concentration-dependent and tannin-dependent manners with some differences between the bacterial strains used to trigger hemolysis. In the presence of both *S. aureus* strains, T3 and T4 demonstrated the weakest effect. The logistic equation fitting the data revealed the IC_50_ values for T3 and T4 to be respectively 90.6 ± 22.7 µM and 59.5 ± 9.6 µM for the NCTC 5655 strain and respectively 52.2 ± 1.2 µM and 60.4 ± 3.3 µM for the 8325-4 strain. The strongest anti-hemolytic effects were displayed by the tannins T1 and by T2. The IC_50_ values were slightly lower in case of hemolysis caused by the NCTC 5655 strain compared to the 8325-4 strain, they were 7.26 ± 0.29 µM vs. 8.70 ± 0.81 µM for T1, respectively, and 12.96 ± 1.18 µM vs 22.35 ± 2.80 µM for T2, respectively. However, the protective effect of the tannins stagnated at 12 ± 3% of hemolysis at the highest tannin concentration in case of the NCTC 5655 strain while the hemolysis triggered by the 8325-4 strain was almost completely abolished (only 4 ± 2% of the remained hemolysis). The more pronounced antihemolytic effect of T1 and T2 at the highest concentration against strain 8325-4 is apparently associated with their inhibitory activity relative to other cytolysins produced by this strain. Overall, these results suggest that T1 had the strongest anti-hemolytic effect at micromolar concentrations.

The antimicrobial activity of tested tannin against both strain 8325-4 and NCTC 5655 was estimated as the lowest concentration of tannins that results in microbial death (MBC). As presented in Table 1 tannins exhibit different effects with respect to both strains. The most active again was the T1 compound with the lowest MBC with respect to NCTC 5655 (25 μM) and 8325-4 (50 μM), while the weakest effect shown T4 and MBC for both strains was 100 μM.

**Table 1:** Antimicrobial activity of the tannins T1–T4.

Compound	Bacterial strain	
	*S. aureus* NCTC 5655	*S. aureus* 8325-4
**Minimal bactericidal concentration MBC [μM]**		
T1	25	50
T2	50	75
T3	100	100
T4	75	75

In connection with these data it cannot be excluded that the antihemolytic activity of the tannins studied on whole bacteria was also related to their action on the bacteria metabolism, which could lead to a decrease in the amount of toxins released, similar to that shown for (-)-epicatechin gallate^9^. Therefore, next we studied the tannin effect on hemolysis caused by commercially available *S. aureus* αHL, a water-soluble 293-amino acid monomeric polypeptide. The binding of seven αHL monomers to a target cell leads to their oligomerization and formation of a nonlytic prepore with subsequent transition to the transmembrane channel as a result of further penetration of subunits into the membrane^53^. The membrane pore formation by αHL causes cytolysis of erythrocytes and of other cells^54^.

**Figure 5:**
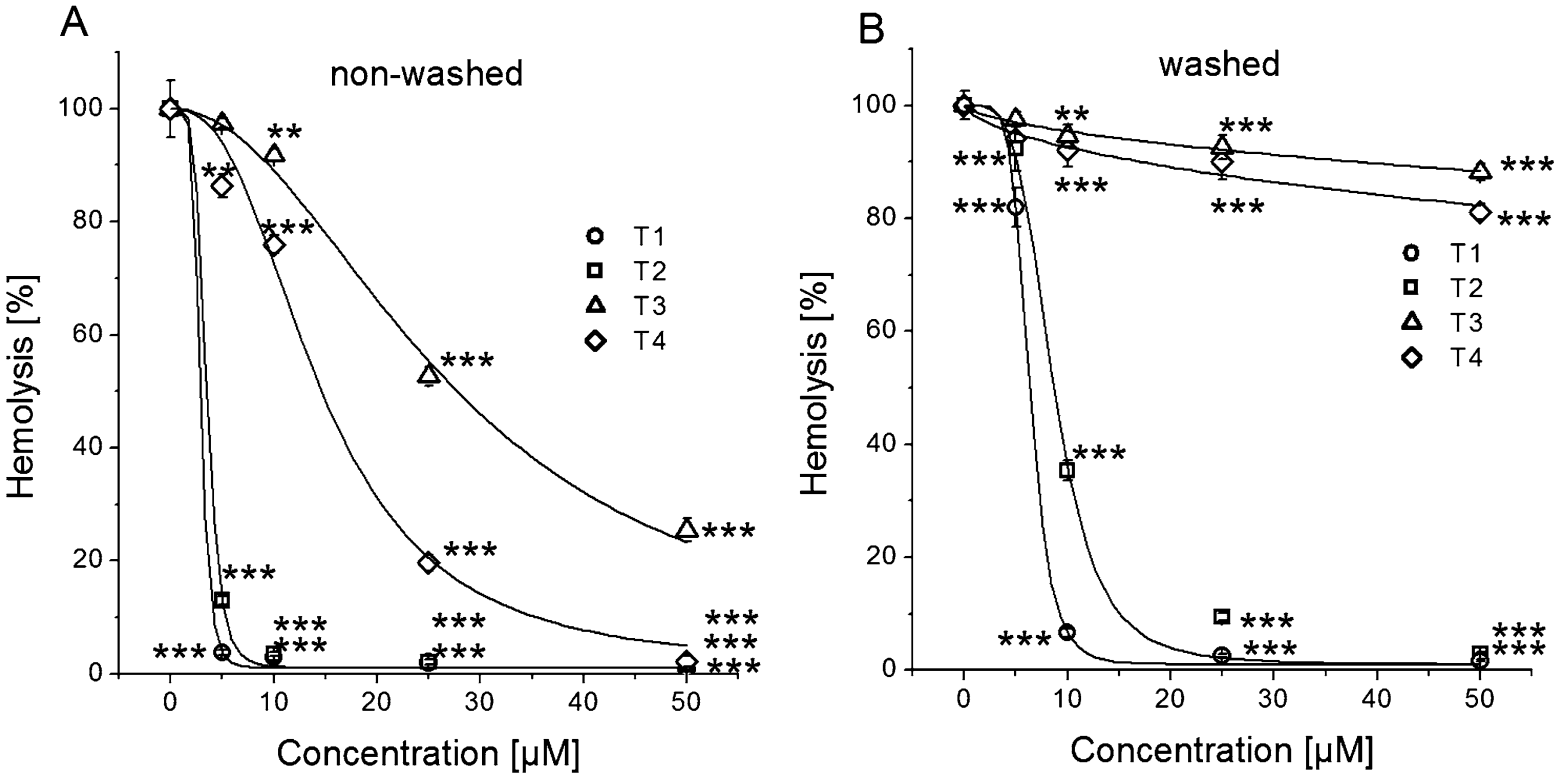
Effects of tannins against hemolysis of sheep erythrocytes induced by α-hemolysin at different incubation conditions. (**A**) Erythrocytes were preincubated with tannins and tannins were also present during incubation with α-hemolysin. (**B**) Erythrocytes were first preincubated with tannins and then washed and thus were absent in the incubation media containing α-hemolysin. The data presented are the means ± SD, n = 6. Lines represent best fit of logistic equation to the data. The effects of the tannins were statistically significant according to one-way ANOVA test, * < 0.05, **p < 0.01, ***p < 0.001.

Exposure of erythrocytes to 50 nM αHL resulted in 62.72 ± 7.74% hemolysis, which in this case was taken as 100%. The results clearly show that all the four tested compounds prevented hemolysis, but their effectiveness varied (Fig. 5A). In general, all the compounds were able to strongly prevent hemolysis at the high concentrations, and much lower compound concentrations were needed to cause half-maximal inhibition of hemolysis. The logistic equation fitting the data revealed the IC_50_ values to be 2.94 ± 1.45 µM, 3.52 ± 1.37 µM, 14.52 ± 1.10 µM and 27.47 ± 1.06 µM for T1, T2, T4 and T3, respectively. It follows that the compounds suppressed hemolysis induced by αHL with the same efficiency sequence depending on their molecular structures as in the study using whole bacteria.

Since the experimental conditions included preincubation of erythrocytes with the tannins and then their continuous presence during hemolysis triggering, the antihemolytic activity of tannins can be also associated with partial neutralization of the toxin itself or/and with modification of physicochemical properties of membranes, which could lead to a restriction of toxin interaction with the membrane. In order to exclude the interaction of the tannins with αHL during incubation, we next studied the antihemolytic activity of the compounds on erythrocytes which were first preincubated with tannins and then subsequently washed.

The data obtained (Fig. 5B) clearly show a relationship between the strength of interaction of the tannins with erythrocyte membrane and their antihemolytic activity. Washing of erythrocytes doubled the half-maximal effect concentrations for T1 and T2: the IC_50_ values raised to 6.37 ± 0.79 µM and 8.68 ± 0.40 µM, respectively (compared to 2.94 ± 1.45 µM and 3.52 ± 1.37 µM in non-washed erythrocytes). These results indicate that T1 and T2 bound strongly to the membrane in a concentration-dependent manner and even after washing could prevent hemolysis. In the case of T1 and T3, there was observed much less protection of erythrocytes against αHL—induced hemolysis compared to unwashed erythrocytes. The IC_50_ values increased approximately 40-fold to 590 ± 354 µM and 1,184 ± 489 µM for T4 and T3, respectively (vs 14.52 ± 1.10 µM and 27.47 ± 1.06 µM in non-washed erythrocytes). This indicates very poor binding of these two tannins to the erythrocyte membrane.

The above results clearly demonstrate that even after washing off the tannins from pretreated erythrocytes, a certain amount of the compounds remained bound to the membranes and apparently changed their properties, thus attenuating manifestation of hemolytic activity of the αHL. To understand the influence of tannins on the erythrocyte membrane better, the effect of the compounds on the structure of erythrocyte membranes and the membrane zeta-potential (ZP) were studied.

### Influence of tannins on the structure, the surface charge density and zeta potential (ZP) of erythrocytes membrane

**Figure 6:**
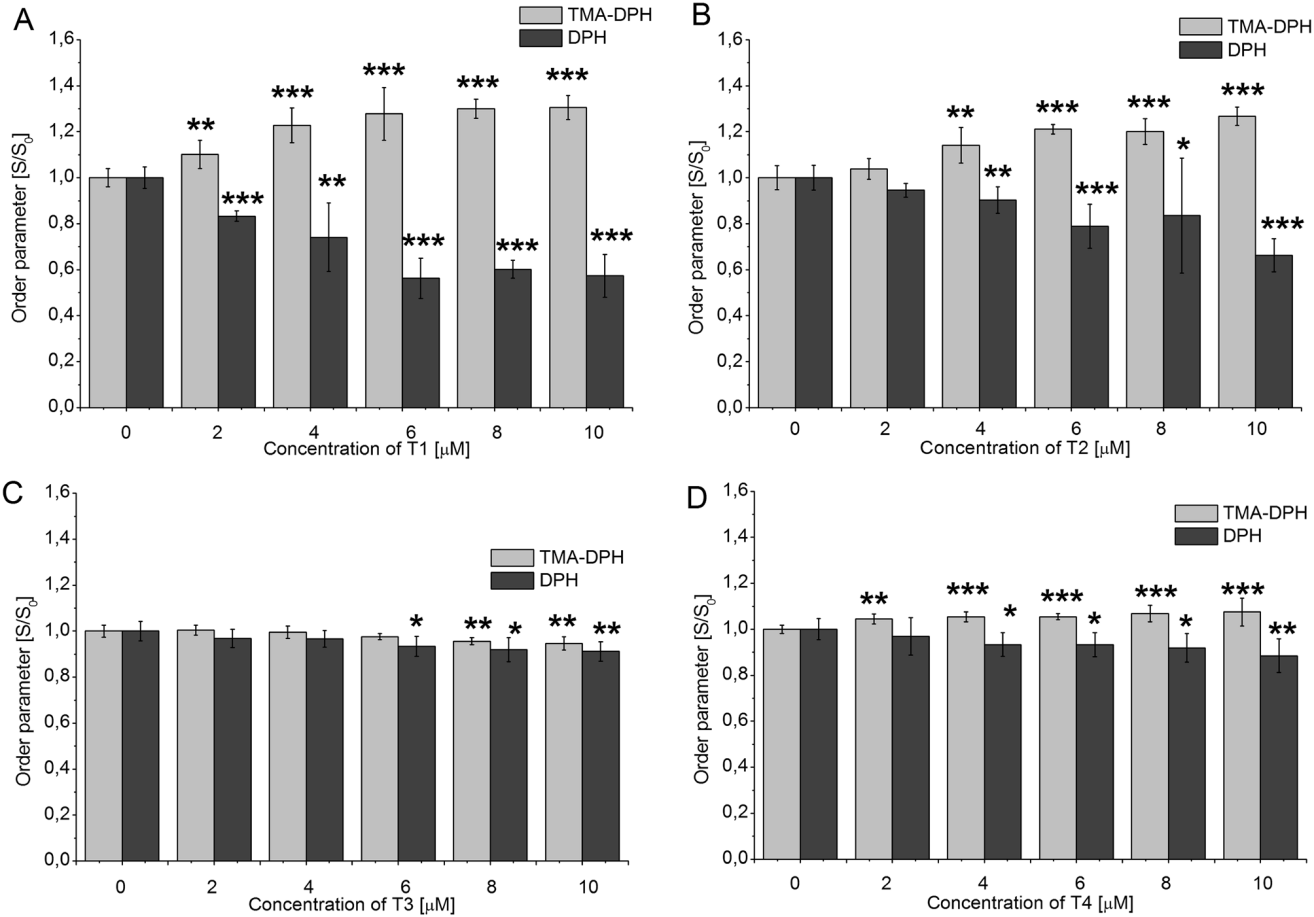
Changes of the order parameter S/S_0_ of erythrocytes membrane in the presence of tannins. S is sample with tannin, S_o_ is sample without tannin. The data presented are the means ± SD (n = 6). The effects of the tannins were statistically significant according to one-way ANOVA test, * < 0.05, **p < 0.01, ***p < 0.001.

We examined the effect of the tannins on lipid ordering in the erythrocyte membrane at different depth of the lipid bilayer by measuring the fluorescence anisotropy values for TMA-DPH and DPH probes that differ in localization in the membrane. The TMA-DPH probe is located in the outer area of the membrane at the level of the fourth carbon atom while DPH is inserted in the membrane hydrophobic region in the middle of the lipid bilayer. The values for fluorescence anisotropy were used to calculate the lipid order parameter^55^. The results are presented as the ratio (S/S_0_) of the values of the order parameter in the presence of the tannins (S) to those in their absence (S_0_) (Fig. 6).

Using the TMA–DPH probe we showed that T1 and T2 increased the order parameter in a concentration-dependent manner. At the highest concentration of 10 μM, the parameter value increased from 1 (control value without tannins) to 1.305 ± 0.053 for T1 and to 1.267 ± 0.0398 for T2, which indicates stiffening of the outer area of the membrane. The effects of T4 and T3 were less pronounced, but still statistically significant. At the highest concentration of 10 µM, the value of the order parameter increased to 1.074 ± 0.059 for T4, while in case of T3, it decreased to 0.947 ± 0.028. Measurements carried out using the DPH probe showed that T1 and T2 at the highest concentration of 10 μM reduced the value of the order parameter to 0.574 ± 0.092 and 0.662 ± 0.072, respectively. This demonstrates an increase in the fluidity of hydrophobic area. T3 and T4 at the same concentration acted much weaker, but also reached the statistically significant value of the order parameter at 0.911 ± 0.042 T3 and 0.885 ± 0.073 for T4. Overall, these results reveal stiffening of the membrane area near its surface and fluidification of the hydrophobic membrane proper in the presence of the studied tannins.

**Figure 7:**
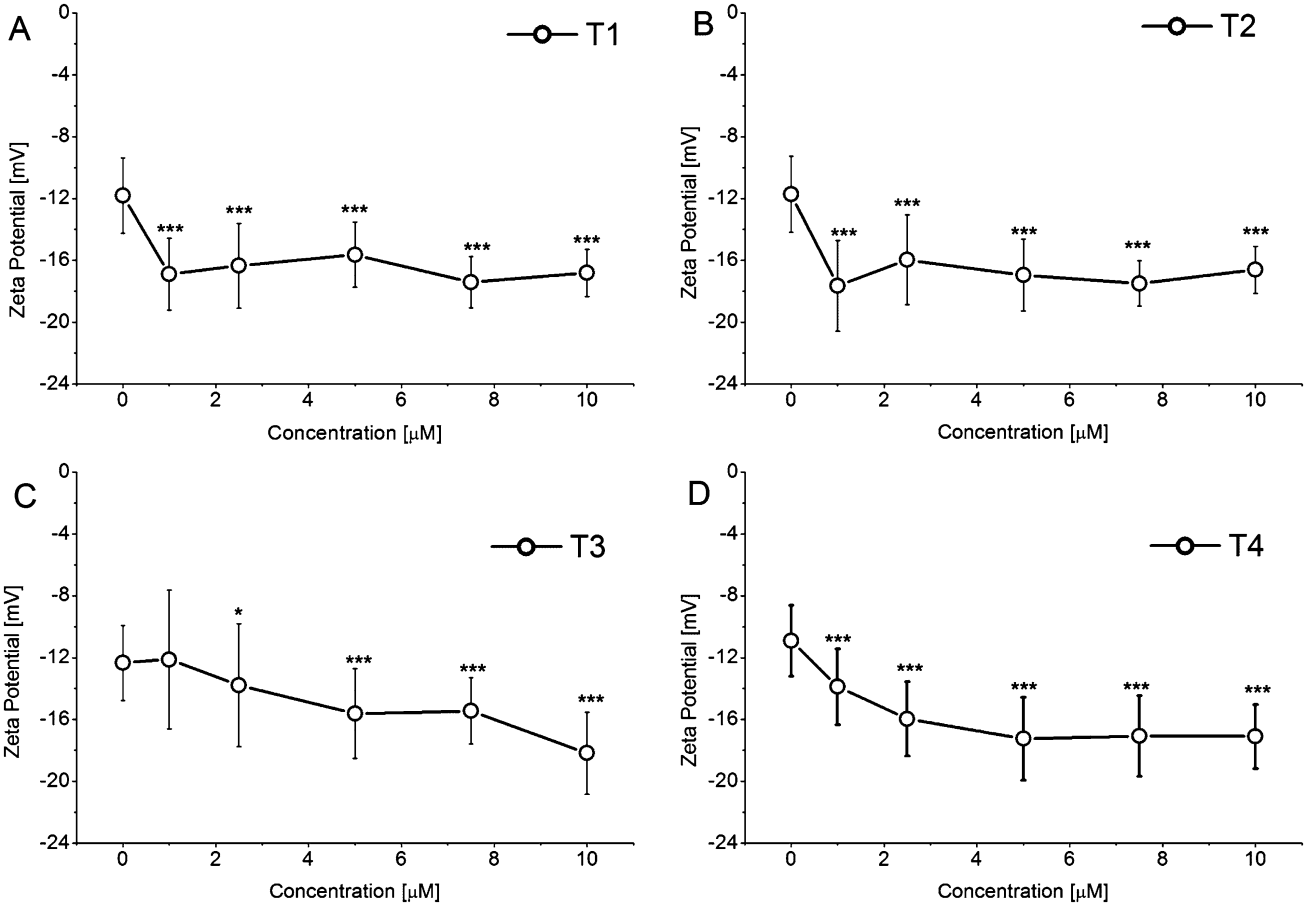
Changes of erythrocytes Zeta potential in the presence of tannins. The data presented are the means ± SD (n = 6). The effects of the tannins were statistically significant according to one-way ANOVA test, * < 0.05, **p < 0.01, ***p < 0.001.

The erythrocyte membrane contains glycoproteins which impart a negative charge to the surface of red blood cells due to the presence of a carboxyl group of sialic acids. As a result of the electrostatic interaction between the negative charge of the erythrocyte membrane and ions of the opposite charge in the surrounding medium, a double layer is formed which generates a potential called the zeta potential (ZP). ZP is an important parameter for stabilization of erythrocytes since it prevents aggregation of cells between themselves and their adhesion to cells of vascular walls^56^. The results of ZP measurements (Fig. 7) showed differences in the effects of the tested tannins.

In the presence of T2 and T1 at the lowest concentration (1 µM), the greatest negative potential increase from − 11.73 ± 2.46 mV to − 17.64 ± 2.93 mV for T2 and from − 11.82 ± 2.44 mV to − 16.89 ± 2.31 mV for T1 was observed. The subsequent increase in tannin concentration led to a slight decrease of the potential value and at higher concentrations a plateau was observed. T3 and T4 caused a progressive increase in the negative potential value with rising concentrations and at 10 µM this potential reached the value of − 18.18 ± 2.66 mV and of − 17.10 ± 2.08 mV, respectively. The changes in surface charge density strictly followed the evolution of ZP. The maximum increase in negative charge density was observed even at low concentrations of T1 and T2 while this negative charge density increased progressively in the presence of rising concentrations of T3 and T4 (Table 2).

**Table 2:** Dependence of erythrocyte’s membrane surface charge density upon addition of T1–T4.

Concentration (µM)	Compound			
	T1	T2	T3	T4
**Surface charge density (10**^**–2**^** C/m**^**2**^**)**				
0	− 1.77 ± 0.37	− 1.76 ± 0.37	− 1.85 ± 0.36	− 1.64 ± 0.34
1	− 2.53 ± 0.35***	− 2.65 ± 0.44***	− 1.82 ± 0.67	− 2.08 ± 0.37***
2.5	− 2.45 ± 0.41***	− 2.40 ± 0.44***	− 2.07 ± 0.60*	− 2.39 ± 0.36***
5	− 2.35 ± 0.32***	− 2.54 ± 0.35***	− 2.35 ± 0.44***	− 2.59 ± 0.40***
7.5	− 2.61 ± 0.25***	− 2.63 ± 0.22***	− 2.32 ± 0.32***	− 2.56 ± 0.39***
10	− 2.52 ± 0.23***	− 2.49 ± 0.23***	− 2.73 ± 0.40***	− 2.57 ± 0.31***

Statistical significance was estimate using One-Way ANOVA, *p < 0.05; ***p < 0.0001, results compared to control.

## Discussion

Tannins are secondary metabolites of plants, characterized by a wide range of biological effects as anticancer, anti-inflammatory, anti-mutagenic, anti-platelets, anti-bacterial, and antiviral activities^38,57^. The antibacterial effects of tannins are widely studied on purified plant extracts. However, little attention has been paid to a structure—functional analysis of these compounds. At the same time, tannins are characterized by great structural diversity and, therefore, different antibacterial activities. For example, it has been established that antivirulence activity relative to staphylococcal enterotoxin A depends on the degree of polymerization of procyanidins. It has been shown that high molecular procyanidins bind to the toxin and inhibit its activity while low molecular ones inhibit only its production^58^.

Other studies showed that polyphenols containing two or more galloyl groups as gallocatechin gallate (GCG), epigallocatechin gallate (EGCG) and pentagalloylglucose (PGG) inactivated *Escherichia coli* heart labile toxin by formation of high molecular aggregates, PGG having 5 galloyl groups manifesting the greatest effect^16^.

The structure—functional dependence of the antibacterial activity of polyphenols relative to *S. aureus* was best studied for flavonoids and catechins.

A comparative analysis of the influence of catechins, their galloylated derivatives and tannic acid on plasma coagulation by *S. aureus* revealed a correlation between the content of galloyl groups in their molecules and the inhibitory effect^41^.

High antihemolytic activity and specificity of action in relation to *S. aureus* αHL and *Vibrio cholerae* O1 hemolysin were also shown for galloylated catechins^59^.

In fact, the dependence of antihemolytic activity with respect to αHL of *S. aureus* on the molecular structure was demonstrated for a number of flavonoids^60,61^.

In our work, we showed the antihemolytic activity of four tannins relative to two strains of *S. aureus* (8325-4 and NCTC 5655) as well as αHL, and revealed that the degree of reduction of hemolysis and their interaction with erythrocyte membrane were not uniform and strongly depended on their structure (Fig. 2).

We consider that the antihemolytic effect, as one of the mechanisms, was associated with an increase in the ordering of the structure of the outer layer of erythrocyte membranes, which, in turn, apparently limited the incorporation of the hemolysin monomer into the membrane and / or αHL splicing into the heptamer necessary for pore formation.

The smallest antihemolytic effect was exhibited by tannins containing a valoneoyl group (T3, T4) that is a limitation factor of the rotational mobility of the aromatic rings^46^. In addition, these tannins have a carboxyl group which, as a result of ionization via deprotonation in solution, can restrain the interaction of the compounds with negatively charged erythrocytes.

A correlation between the antihemolytic activity and the structural changes induced by them in the membrane was shown for tannins without a valoneoyl group (T1, T2), which is apparently connected with their more intensive binding to membranes, as was demonstrated by experiments with washed erythrocytes after their pretreatment with the compounds.

It is known that tannins may also form aggregates with αHL, inhibiting its action on erythrocytes as was shown by Choi et al.^62^. However, the same authors demonstrated that the antihemolytic effect of tannins isolated from apple and hop bract was observed if erythrocytes pretreated with the tannins were washed before addition of *S. aureus* αHL. These data are consistent with ours (Fig. 5) and confirm the implementation of the antihemolytic activity of tannins also at the level of erythrocyte membranes.

As noted above, tannins interact strongly with proteins, including membrane ones. It is known that tannins-proteins reactions involve hydrophobic interactions between polyphenol aromatic rings and hydrophobic residues of amino acids, mainly proline pyrrolidine rings, and hydrogen bonds are also formed between the hydroxyl groups of polyphenols and the acceptor site for hydrogen ions in the proteins^63^. Moreover, as has been shown recently, the interaction of tannins with membrane lipids plays an important role in the manifestation of their biological activity. Tannins can change dynamic parameters and physicochemical properties of membranes^64^ and also form lipid rafts, i.e., heterogeneous membrane structures that play an important role in regulating physiological processes in the cell^65,66^.

Both phosphate and –CH_2_ groups of lipid aliphatic chains and hydroxyl groups of tannins are involved in the interaction between lipids and the tannins^65,67,68^.

It is known, the interaction of αHL with membranes occurs at the level of the phosphatidylcholine head group^69,70^ and/or the ADAM10 protein receptor in the case of red blood cell highly sensitive to the toxin, such as rabbit erythrocytes^54^.

In our experiments, we used sheep erythrocytes for which no specific receptor for αHL was found, which means that in this case, the interaction of the toxin occurs at the level of the phosphatidylcholine head and the occupation by tannins studied of the α-HL binding sites on the membrane can also lead to a restriction of the interaction with the toxin. It can be assumed that in the case of rabbit erythrocyte, the antihemolytic effect of the studied tannins could be stronger due to the interaction with lipids as well as the protein receptor.

The relationship between the change in the ordering parameter of the membranes and ZP in the presence of the tannins should also be noted. T1 and T2 increased the ordering parameter more strongly even at low concentrations therefore they dramatically changed the ZP. This suggests that the presence of a greater amount of gallic acid residues in T1 and T2 allows them to penetrate deeper into the hydrophobic core of erythrocyte membranes. At higher concentrations of these compounds, interactions between the groups on the surface of the membrane and the groups of –OH tannin molecules may also occur. T3 and T4 showed poor binding to the erythrocyte membrane (smaller parameter S change) consequently the ZP decreased with the concentration.

The tannins used in this study differ not only in the presence of the valoneoyl group but also in the MM, i.e. in the number of both aromatic rings and hydroxyl groups. T1 has 7 gallic acid residues and 19 –OH groups while T2 has 5 gallic acid residues and 15 –OH groups. T4 has 15 hydroxyl groups and 5 aromatic rings, while T3 is composed of 4 aromatic rings and 13 hydroxyl groups. T3 and T4 contain valoneoyl groups at C1 and C2 positions and at C4 and C6, respectively.

It is believed that tannins with higher MM and hence a larger number of functionally active groups (aromatic rings and OH–group) interact stronger with biomolecules, although it should be noted that there is evidence contrary to this dogma.

It was shown that smaller 1-*O*-galloyl-4,6-hexahydroxydiphenoyl-β-d-glucose possessing 11 OH-groups and 3 aromatic rings interacted with albumin more strongly in comparison with much bigger bihexahydroxydiphenoyl-trigalloylglucose (20 OH-groups and 7 aromatic rings)^71^. This difference can be due to the presence of the two hexahydroxydiphenoyl groups in the bihexahydroxydiphenoyl-trigalloylglucose molecule, which may limit the flexibility of the molecule. This conclusion is consistent with the data of Hofmann et al.^43^, who described that more flexible PGG (gallotannin) forms precipitates with albumin with higher efficiency compared to castalagin and grandinin containing hexahydroxydiphenoyl groups (ellagitannins).

The comparison in all our experiments of the antihemolytic and membrane-modifying action on erythrocytes of two tannins varying in MM (T1 and T2), but not having hexahydroxydiphenoyl and valoneoyl groups in their structure, showed that the compound with a large number of OH and aromatic rings (T1) has the greatest effect, which is consistent with the generally accepted concept. However, the comparison of the activities of T2 and T4 having an equal number of aromatic rings and OH groups (5 and 15, respectively) showed that T4 possessed significantly lower activity. T4 is rather stiff in comparison with T2 due to the presence of the valoneoyl group at C4 and C6 positions in the molecule, which can limit its spatial reorganization and ability to interact with the membrane. Our assumption is in accordance with the work of Beretta et al.^72^, which on the basis of studies of the interaction of PGG with liposomes by NMR and theoretical calculations, proposed a model that implied a gradual spatial reorientation of all of the 5 residues of PGG gallic acid interacting with lipids. The comparison of the activities of two tannins containing a valoneoyl group (T3 and T4) but differing in MM showed the strongest effect of the tannin having an additional gallic acid residue and, accordingly, 3-OH groups, i.e., of the tannin with a large MM.

The obtained results indicate that the interaction of primarily OH-groups of tannins with phosphate heads of phosphoslipids (mainly phosphatidylcholine as the main component of the membranes) results in the stiffening of erythrocyte membranes and the restriction of lateral diffusion, both leading to the limitation of formation of a functional channel by αHL. This interaction depends not only on the number of OH– groups in molecules of tannins but also on the availability of OH—groups to membrane phospholipids, in other words on the rotational mobility of gallic acid residues containing these groups i.e. on the flexibility of the molecule. We have shown that tannins containing a valoneoyl group in which gallic acid residues have limited rotational mobility interact weaker with erythrocytes membranes (they less potently change the membrane ordering parameter) and exhibit the least antihemolytic effect. We suggest also that the studied tannins can directly interact with secreted αHL modifying its structure and activity. Alternatively, tannins can also change or block the conductivity of channels already formed by αHL, similar to what has been shown for compounds of plant^27^ and non-plant nature^73,74^. Preliminary electrophysiological studies in bilayer lipid membranes and protein spectroscopy studies performed for T2 and T4 confirm this hypothesis.

## Conclusion

The studied tannins—3,6-bis-*O*-di-*O*-galloyl-1,2,4-tri-*O*-galloyl-*β*-d-glucose (T1), 1,2,3,4,5-penta-*O*-galloyl-*β*-d-glucose (T2), 3-*O*-galloyl-1,2-valoneoyl-*β*-d-glucose (T3), and 1,2-di-*O*-galloyl-4,6-valoneoyl-*β*-d-glucose (T4)—exhibited antihemolytic activity against two strains of *S. aureus* (8325-4 and NCTC 5655) and αHL, and that this effect correlated with the increased rigidity of the outer layer of erythrocyte membranes. These data suggest that structural changes in erythrocyte membranes caused by the tannins limited the binding and/or oligomerization of the toxin. The difference in antihemolytic and the membrane-modifying activities of the tannins was related to their structures. The compounds containing a valoneoyl group which limited their rotational mobility were less active.

## Methods

### Bacterial strains and growth conditions

*S. aureus* strains 8325-4 and NCTC 5655, obtained from Prof. Jan Oscarsson (University of Lund, Sweden) and National Collection of Type Cultures (UK), respectively, were used in the study. Bacteria were grown overnight at 30 °C in Mueller Hinton (MH) broth with shaking at 200 rpm or on nutrient agar (NA) plates which were obtained from Oxoid (Basingstoke, UK).

### Materials

The compounds: 3,6-bis-*O*-di-*O*-galloyl-1,2,4-tri-*O*-galloyl-*β*-d-glucose (T1), 1,2,3,4,5-penta-*O*-galloyl-*β*-d-glucose (T2) from leaves *Rhus typhina* L., 3-*O*-galloyl-1,2-valoneoyl-*β*-d-glucose (T3) and 1,2-di-*O*-galloyl-4,6-valoneoyl-*β*-d-glucose (T4) from roots *Euphorbia helioscopia* and *Euphorbia jaxartica* Prokh (Fig. 2) were isolated and characterized. 1,6-diphenyl-1,3,5-hexatriene (DPH) and 1-(4-trimethylammoniumphenyl)-6-phenyl-1,3,5-hexatriene (TMA-DPH), αHL were received from Sigma-Aldrich (St. Louis, MO). All other reagents were purchased from POCH (Poland). Sheep blood was purchased from the GrasoBIOTECH (Graso Company, Poland).

### Tannins isolation and characterization

Source material for tannin isolation (roots of plants belonging to the family *Euphorbiaceae* (Spurge) and leaves of *Rhus typhina* L. from the family *Annacardeaceae*) was collected in Uzbekistan at the end of vegetation period. Tannins were isolated as described earlier^75,76^. Briefly, dried and crushed plant material was treated with chloroform to remove lipophilic substances. The chloroform was removed by filtration and material was dried and then extracted with 70% aqueous acetone. The acetone was evaporated under vacuum and the aqueous residue was treated with ethyl acetate. The ethyl acetate extract was concentrated under vacuum followed by addition of a four-fold volume of hexane to obtain total fraction of polyphenols. Silica gel column chromatography using chloroform and mixture methanol-chloroform (2:8) as a solvent phase was applied to the total extracted polyphenols to obtain some polyphenol fractions including tannin one. In order to isolate individual compounds, fractions containing hydrolysable tannins were rechromatographed on the silica gel column (L40/100) with various solvent systems. One was a gradient of diethyl ether:ethyl acetate starting with the ratio 2:8 and ending by pure ethyl acetate and second system was 60% of aqueous methanol. Purities of compounds were between 95 and 98%.

Based on ^1^H-NMR, ^13^C-NMR and UV–Vis spectroscopy techniques, the chemical parameters for used tannins were obtained. The characteristics of the studied tannins with information on the original plant material are presented in Supplementary Information S1 Appendix.

### Antimicrobial activity

The minimum bactericidal concentration (MBC), the lowest concentration of tannin required to kill bacterial cells was determined using p-iodonitrotetrazolium chloride (INT). The colorless INT acts as an electron acceptor and living bacteria reduce it to a red-coloured formazan product^77^. Briefly, the *S. aureus* strains NCTC 5655 and 8325-4 maintained in MH broth were centrifuged (800 × g, 5 min). After removal of the supernatant, the bacterial cells were resuspended in 0.9% NaCl and adjusted to absorbance A = 2.4 at a wavelength λ = 600 nm. Next, 50 µl bacterial suspension were added to the wells of a sterile 96-well microtiter plate containing 150 µl 0.9% NaCl and different concentration of tannins in range of 10–500 µM. The control wells did not contain tannins. The plate was shaken on microplate shaker for 1 min and incubated for 24 h at 37 °C. In order to detect living bacteria, 40 µl of 0.4 mg/ml INT was added to wells and incubated for 30 min at 37 °C. After incubation, the wells containing only bacteria had red color, while in wells where bacterial were killed by tannins the solution was much fainter. The lowest concentration of tannin showing no red colour was taken as its MBC.

### Hemolysis assay

The effect of *S. aureus* NCTC 5655 and 8325–4 on the integrity of sheep erythrocytes was detected basing on the Olchowik-Grabarek et al. method with some modifications^22^. Shortly, sheep blood was centrifuged (2,655 × g, 15 min, 4 °C) and plasma and buffy coat were removed by aspiration. Erythrocytes were washed twice with 0.9% NaCl, and then 1% suspension was prepared in 0.9% NaCl. 2 ml of 1% suspension of erythrocytes was incubated 30 min at 37 °C in the presence or absence of tannins. Then 100 µl of bacteria in MH broth (A_620_ = 2.4) was added to each sample. After incubation for 60 min at 37 °C, 0.5 ml of suspension was taken from every sample and mixed with 1 ml of 0.9% NaCl. For obtaining of 100% hemolysis 1 ml of water was added to the control sample. All the samples were centrifuged, and absorbance of supernatants was measured using Jasco V-770 spectrophotometer (Japan) at 540 nm.

In the study of the αHL—induced hemolysis, 2 ml of 1% suspension of sheep erythrocytes in 0.9% NaCl was incubated with and without tannins for 30 min. Then the suspension was incubated for 45 min with αHL (50 nM final concentration). All the samples were centrifuged, and absorbance of supernatants was measured at 540 nm. Obtained results are presented as percent of hemolysis depending on the concentration of tannins.

### Tannin—membrane interaction studies, fluorescence spectroscopy

The erythrocyte membrane ordering parameter was estimated by using a steady—state fluorescent polarization technique. The suspension of erythrocytes (2 ml of 0.05% hematocrit in 0.9% NaCl) was labelled with a fluorescent probe (DPH or TMA-DPH, respectively) at a concentration of 1 μM (10 min, 37 °C). The fluorescence measurements were carried out at 37 °C using a Perkin-Elmer LS-55 (Perkin-Elmer, UK) spectrofluorometer equipped with a fluorescence polarization device. The readings were taken at intervals of 2 s. Changes in membrane fluidity after addition of tannins in the concentration range of 2–10 µM were determined based on polarization values of the samples (r).

The polarization values (r) were calculated by the fluorescence data manager program using the Jablonski equation:

$$r=\frac{{I}_{VV}-G{I}_{VH}}{{I}_{VV}+2G{I}_{VH}}$$

where I_*VV*_ and I_*VH*_ are the vertical and horizontal fluorescence intensities, respectively to the vertical polarization of the excitation light beam. The factor G = I_HV_/I_HH_ (grating correction factor) corrects the polarizing effects of the monochromator. The excitation wavelengths were 348 nm (DPH) and 340 nm (TMA-DPH) and the fluorescence emission was measured at 426 nm for DPH and 430 nm for TMA-DPH^78^.

Based on the data obtained, the membrane ordering parameter was calculated using the equation^47^:

$$S=\frac{\sqrt{\left[1-2\left(\frac{r}{{r}_{0}}\right)+5{\left(\frac{r}{{r}_{0}}\right)}^{2}\right]}-1+\frac{r}{{r}_{0}}}{2\left(\frac{r}{{r}_{0}}\right)}$$

where *r*_*0*_ is the fluorescence anisotropy of DPH or TMA-DPH in the absence of any rotational motion of the probe. The theoretical value of *r*_*0*_ of DPH and TMA-DPH is 0.4.

### Assessment of surface charge density and ZP of erythrocytes membrane

The erythrocyte ZP was determined based on erythrocytes electrophoretic mobility in 1 ml of a 0.001% cell suspension in 0.9% NaCl. After 30 min incubation of cells with tannins (1, 2.5, 5, 7.5, 10 µM), samples were placed in a special chamber and electrophoretic mobility was measured in a Zetasizer Nano ZS (Malvern Instruments, Malvern, UK). The electrophoretic mobility values obtained were used to calculate the ZP according to the Smoluchowski’s equation:

$$\xi =\frac{3\mu \eta }{2\varepsilon {\varepsilon }_{0}f\left(\kappa a\right)}$$

where *μ* is the electrophoretic mobility, *η* is the solution viscosity, *a* is the erythrocytes radius, *κ − *1 is the Debye length, *ε*_0_ and *ε* are the permittivity of free space and the relative permittivity of the medium, respectively.

On the basic electrophoretic mobility data were also calculated the surface charge density values using the equation:

$$\delta =\frac{\mu \eta }{d}$$

where *μ* is the electrophoretic mobility, *η* is the solution viscosity, *d* is the diffuse layer thickness that was calculated from equation:

$$d=\sqrt{\frac{\varepsilon {\varepsilon }_{0}RT}{2{F}^{2}I}}$$

where R is the gas constant, T is temperature, F is the Faraday number, I is the ionic strength of 0.9% NaCl, ε·ε_o_ is the permeability of the electric medium^79^.

### Statistical analysis

The results are presented as mean ± SD. The level of significance was analyzed using one-way ANOVA test. *p* < 0.05 and below was accepted as statistically significant. Statistical analysis was performed using Origin 8.5.1 software (Microcal Software Inc., Northampton, MA).

## Supplementary information

Supplementary information
