# Supplementary material for: Inhibition of interaction between Staphylococcus aureus α-hemolysin and erythrocytes membrane by hydrolysable tannins: structure-related activity study
Source: Sci Rep. 2020 Jul 7;10:11168. doi: 10.1038/s41598-020-68030-1 (PMC7341856; doi:10.1038/s41598-020-68030-1)
Supplement: Supplementary file 1 — Supplementary information [file 41598_2020_68030_MOESM1_ESM.docx]

Supplementary Information

**Inhibition of interaction between *Staphylococcus aureus* α-hemolysin and erythrocytes membrane by hydrolysable tannins: structure-related activity study**

Ewa Olchowik-Grabarek^1^, Szymon Sekowski^1^, Maciej Bitiucki^1^, Izabela Dobrzynska^2^, Vadim [Shlyonsky](https://www.scopus.com/authid/detail.uri?authorId=6602624532&amp;eid=2-s2.0-84941773388)^3^, Maksim Ionov^4^, Paweł Burzynski^1^, Anna Roszkowska^1^, Izabela Swiecicka^1,5^, Nodira Abdulladjanova^6^, Maria Zamaraeva^1^*

*^1^ Department of Microbiology and Biotechnology, Laboratory of Molecular Biophysics, Faculty of Biology, University of Bialystok, Konstanty Ciolkowski Street 1J, 15-245 Bialystok, Poland*

*^2^ Laboratory of Bioanalysis, Faculty of Chemistry, University of Bialystok, Konstanty Ciolkowski Street 1K, 15-245 Bialystok, Poland*

*^3^ Department of Physiology and Pharmacology, Université libre de Bruxelles, 808 Route de Lennik 808, CP604, 1070 Brussels, Belgium*

*^4^ Department of General Biophysics, Faculty of Biology and Environmental Protection, University of Lodz, Pomorska Street 141/143, 90-236 Lodz, Poland*

*^5^ Department of Microbiology and Biotechnology, Laboratory of Applied Microbiology, Faculty of Biology, University of Bialystok, Konstanty Ciolkowski Street 1J, 15-245 Bialystok, Poland*

*^6^ Institute of Bioorganic Chemistry, Academy of Sciences of the Republic of Uzbekistan, Abdullaev Street 83, 100143, Tashkent, Uzbekistan*

*corresponding author Maria Zamaraeva e-mail: m.zamaraeva@uwb.edu.pl

**S1 Appendix.** The characteristics of the studied tannins with information on the original plant material.

**3,6-bis-*O*-di-*O*-galloyl-1,2,4-tri-*O*-galloyl-*β*-D-glucose,** isolated from leaves of *Rhus typhina* L., С_55_Н_40_О_34_, MS m/z: 1243 [M-H]^-^, UV (λ_max_, МеОН, nm): 226, 276. IR (ν_max_, cm^-1^): 3350 (OH), 1695 (-COO-), 1615, 1545, 1450 (aromatic ring), 1320 (-С-ОН), 1250, 1040 (-C-O-C). ^1^H NMR (400 MHz, δ, Me_2_СO-d_6_, ppm): 6.39 (1H, d, J=4 Hz, glucose H-1), 5.66 (1H, dd, J=4 Hz, glucose H-2), 6.06 (1H, t, J=10 Hz, glucose H-3), 5.70 (1H, t, J=10 Hz, glucose H-4), 4.60 (1H, m, J=7 Hz, glucose H-5), 4.60 (2H, dd, J=7 Hz, glucose H-6), 6.84, 6.87, 7.10, 7.11, 7.16, 7.17 (2H, s, galloyl group). ^13^C-NMR (100 MHz, acetone-d6+D_2_O, ppm): 96.5 (C-1), 74.2 (C-2), 73.0 (C-3), 69.6 (C-4), 75.1 (C-5), 63.4 (C-6) glucose; 120.4 (С-1), 110.1 (С-2, C-6), 145.9 (С-3, C-5), 139.3 (С-4), 168.8 (С-7) – galloyl group; 115.8 (C-1), 126.4 (C-2), 108.1 (C-3), 145.8 (C-4), 136.4 (C-5), 144.5 (C-6), 168.0 (C-7), 115.5 (C`-1), 125.8 (C`-2), 107.8 (C`-3), 145.8 (C`-4), 136.6 (C`-5), 144.4 (C`-6), 167.6 (C`-7) – digalloyl - groups.

**1,2,3,4,6-penta-*O*-galloyl-*β*-D-glucose**, isolated from leaves of *Rhus typhina* L., C_41_H_32_O_26_, MS m/z 939 [M-H]^-^. UV (λ_max_, МеОН, nm): 222, 268. IR (ν_max_, cm^-1^): 3380 (OH), 1687 (-COO-), 1612 (aromatic ring), 1344 (-С-ОН), 1024, 765 (-C-O-C). ^1^H NMR (400 MHz, δ, Me_2_СO-d_6_, ppm): 6.23 (1H, d, *J* = 8.4 Hz, glc H-1), 5.58 (1H, dd, *J* = 8.4, 9.6 Hz, glc H-2), 5.89 (1H, t, J=8.3 Hz, glc H-3), 5.60 (1H, d, *J* = 8.7, 9.5 Hz, glc H-4), 4.50 (1H, d, *J* = 10.8 Hz, glc H-5), 4.34-4.44 (2H, m, glc H-6), 6.94, 6.98, 7.04, 7.10, (10H, s, Gal.). ^13^C-NMR (100 MHz, acetone-d6+D_2_O, ppm): glucose: 63.1 (C-1), 69.8 (C-2), 72.2 (C-3), 74.1 (C-4), 74.4 (C-5), 93.8 (C-6), galloyl group: 110.4x5, 110.6x5 (C-2, C-6), 119.7, 120.0 x 2, 120.1, 121.0 (C-1), 140.1, 140.2, 140.3, 140.4, 140.7 (C-4); 146.2x2, 146.3x2, 146.4x 2, 146.5x2 (C-3, C-5), 166.2, 166.9, 167.0, 167.3, 167.9 (C-7).

**3-*O*-galloyl-1,2-valoneoyl-*β*-D-glucose** isolated from roots *Euphorbia helioscopia* С_34_Н_26_О_23_, MS *m/z* 801 [M-H]^-^.^.^ UV (λ_max_, МеОН, nm): 220, 270. IR (ν_max_, cm^-1^): 3500 (ОН), 1690 (-СОО-), 1620, 1545, 1450 (aromatic ring), 1320 (-С-ОН), 1250, 1045 (-С-О-С). ^1^H NMR (400 MHz, δ, Me_2_СO-d_6_, ppm): 6.60 (1Н, d, J=4.0 Hz, glc H-1), 5.65 (1H, dd, J=4, 10 Hz, glc H-2), 6.65 (1H, t, J=10 Hz, glc H-3), 3.99 (1H, t, J=10 Hz, glc H-4), 3.39 (1H, m, J=7 Hz, 13, glc H-5), 3.79 (2H, dd, J=13 Hz, glc H-6), 7.18, 7.50, 8.31 (3H, s, J=2 Hz, Val.), 7.12, 7.17 (2Н, s, J=2 Hz, Gal.). ^13^C-NMR (100 MHz, acetone-d6+D_2_O, ppm): galloyl group: 120.6 (C-1), 109.6 (C-2), 144.8 (C-3), 139.4 (C-4), 145.3 (C-5), 109.9 (C-6), 166.2 (C-7); valoneoyl group: 114.4 (C-1), 126.5 (C-2), 106.6 (C-3), 144.9 (C-4), 136.1 (C-5), 144.5 (C-6), 169.5 (C- 7), 116.6 (C-1`), 126.0 (C-2'), 102.6 (C-3`), 146.7 (C-4`), 135.3 (C-5`), 145.0 (C-6`), 168.3 (C-7`), 111.5 (C-1``), 135.5 (C-2``), 140.6 (C-3``), 139.9 (C-4``), 143.0 (C-5``), 110.2 (C-6``), 164.1 (C-7``); glucose: 92.3 (C-1), 75.3 (C-2), 78.0 (C-3), 70.6 (C-4), 72.6 (C-5), 63.6 (C-6).

**1,2-di-*O*-galloyl-4,6-valoneoyl-*β*-D-glucose** obtained from roots *Euphorbia jaxartica Prokh.* С_41_Н_30_О_27_,MS  *m/z* 953 [М-Н]^-^. UV (λ_max_, МеОН, nm): 216, 255, 370. IR-(ν_max_, cm^-1^): 3350 (ОН), 1695 (-СОО-), 1615, 1545, 1450 (aromatic ring), 1320 (-С-ОН), 1250, 1040 (-С-О-С). ^1^H NMR (400 MHz, δ, Me_2_СO-d_6_, ppm): 6.20 (1Н, dd, J=8.0 Hz, glc H-1), 5.58 (1H, dd, J= 10 Hz, glc H-2), 5.30 (1H, t, J=10 Hz, glc H-3), 5.20 (1H, t, J=9.5 Hz, glc H-4), 4.54 (1H, m, J=3,0 Hz, glc H-5), 3.87 (1H, dd, J=12 Hz, glc H-6), 4.60 (1H, dd, J=12 Hz, glc H-6), 6.79, 6.98, 7.13 (3H, s, J=2 Hz, Val.), 7.12, 7.18 (2Н, s, J=2 Hz, Gal.). ^13^C-NMR (100 MHz, acetone-d6+D_2_O, ppm): galloyl group: 119.0 (C-1), 110.0 (C-2), 145.7 (C-3), 139.4 (C-4), 146.0 (C-5), 110.2 (C-6), 162.8 (C-7); valoneoyl group: 116.0 (C-1), 126.2 (C-2), 107.6 (C-3), 144.8 (C-4), 136.4 (C-5), 144.4 (C-6), 168.5 (C- 7), 116.0 (C-1`), 125.7 (C-2 '), 106.0 (C-3`), 146.8 (C-4`), 137.3 (C-5`), 144.8 (C-6`), 169.3 (C-7`), 114.3 (C-1``), 137.6 (C-2``), 140.2 (C-3``), 140.0 (C-4``), 142.7 (C-5``), 110.5 (C-6``), 167.0 (C-7``); glucose: 95.3 (C-1), 76.5 (C-2), 76.9 (C-3), 69.4 (C-4), 73.2 (C-5), 63.2 (C-6).
